# Dielectric Properties of Transformer Resin Under Varying Conditions: Impact on Instrument Transformer Stability and Accuracy

**DOI:** 10.3390/s25051626

**Published:** 2025-03-06

**Authors:** Simone Vincenzo Suraci, Jizhu Jin, Roberto Tinarelli, Lorenzo Peretto, Davide Fabiani, Alessandro Mingotti

**Affiliations:** Department of Electrical, Electronic, and Information Engineering (DEI), University of Bologna, 40136 Bologna, Italy; jizhu.jin2@unibo.it (J.J.); roberto.tinarelli3@unibo.it (R.T.); lorenzo.peretto@unibo.it (L.P.); davide.fabiani@unibo.it (D.F.)

**Keywords:** accuracy, instrument transformers, ageing, temperature, humidity

## Abstract

The accuracy of instrument transformers (ITs) is vital for the accurate measurement of electrical quantities. However, their performance is influenced by various factors during operation, including environmental conditions such as temperature, pressure, and humidity, as well as other factors like positioning, electromagnetic fields, and geometry. Given that IT accuracy is challenging to verify once installed in the field, it is essential to thoroughly understand its performance beforehand. This paper investigates how variations in resin properties affect IT accuracy. Samples prepared with different curing temperatures were subjected to aging tests, which included exposure to temperature and combined temperature–humidity conditions. Throughout the aging process, the dielectric properties of the samples were measured, and their impact on IT accuracy was evaluated. The results clearly demonstrate that the choice of resin properties is critical to ensure reliable IT performance, as improper selection can lead to significant accuracy deviations.

## 1. Introduction

Instrument transformers (ITs) play a critical role in modern power systems by enabling accurate measurement, protection, and control. These devices, including current transformers (CTs) and voltage transformers (VTs), are designed to step down high voltages and currents to safer and measurable levels. By doing so, they facilitate the operation of metering equipment and protective relays, ensuring both the safety of personnel and the reliability of the power system [1]. ITs are integral to maintaining system stability, enabling the accurate monitoring of power quality, energy consumption, and fault conditions.

Over the years, instrument transformers have undergone significant evolution in design and functionality. Traditional inductive ITs have been widely used for decades due to their robustness and accuracy in steady-state conditions. However, advancements in materials, manufacturing techniques, and digital technologies have led to the development of optical and electronic transformers. These modern variants, typically referred to as low-power instrument transformers (LPITs), offer improved bandwidth, compactness, and immunity to electromagnetic interference, making them particularly suited for smart grid applications. Beyond measurement, they also support key activities such as fault detection, grid synchronization, power quality assessment, and system health monitoring [2]. Their evolution reflects the increasing complexity of power systems and the demand for precise real-time data to optimize grid performance and integrate renewable energy sources effectively. The accuracy of ITs is paramount in ensuring the reliable operation of power systems. Accurate current and voltage measurements directly impact the performance of metering devices and protective relays, which rely on precise input data to operate effectively [3]. Inaccuracies can lead to cascading issues: incorrect billing in energy meters, the misoperation of protective schemes, and compromised fault detection. For instance, an overestimated current during normal operation might cause the unnecessary tripping of circuit breakers, while underestimation during faults could delay critical protective actions, potentially resulting in equipment damage or system instability. High accuracy is also essential for maintaining power quality and energy efficiency. With the increasing penetration of distributed energy resources (DERs), power systems are subject to dynamic variations and harmonic distortions. ITs must maintain accuracy under these conditions to support robust power quality monitoring and compliance with standards. Modern applications, such as phasor measurement units (PMUs) in wide-area monitoring systems, also demand highly precise measurements to detect minute changes in system parameters [4]. As the grid transitions toward smarter and more distributed architectures, the role of accurate ITs becomes even more critical in enabling advanced control strategies, optimizing energy management, and fostering grid resilience. For the above reasons, the literature is very vivid in this respect. For example, ref. [5] proposes tests to evaluate the accuracy when high-frequency components are present. In [6,7], the authors addressed the accuracy issue by modelling CTs and a Rogowski coil, respectively. The authors in [8] dealt with a crucial aspect: how CTs impact the accuracy of the line parameter estimation. They used synchronized measurements to run the study. Finally, in [9], the accuracy is estimated via novel emerging technologies like neural networks and machine learning algorithms.

This paper aims to contribute to the direction of ensuring that the accuracy of ITs is maintained in every operating condition. To this purpose, the idea is to investigate the effects of the insulation properties of the material used to build the ITs on the overall accuracy of the device. In the literature, researchers laterally addressed this issue, like in [10], where the authors examine the use of epoxy polyester mix insulation in dry-type current transformers for enhanced durability, while ref. [11] focused on environmentally friendly epoxy-resin-impregnated paper for 110 kV transformers to promote sustainability. In [12], instead, the authors presented techniques to assess the insulation status of high-voltage cast-resin CTs. Ref. [13] investigates the impact of external factors on the frequency-dependent transfer ratio of medium-voltage (MV) resin-cast transformers. As for [14], a comprehensive review of the technology behind current and voltage measurement and insulation systems in instrument transformers is provided. Finally, ref. [15] proposes a novel “ℓ” shape in partial discharge patterns to identify the critical moment for replacing cast-resin current transformers.

These studies delve into the optimization and characterization of epoxy resins for transformer applications, focusing on enhancing their electrical and thermal performance. Ref. [16] investigated the tuning of epoxy nanocomposites for medium-frequency transformers, emphasizing resin optimization and high-temperature performance. Ref. [17] explored the impact of curing conditions on the electrical properties of epoxy resins, highlighting the critical role of processing parameters. Finally, ref. [18] examines how the dielectric relaxation of epoxy resin influences dielectric loss in medium-frequency transformers, aiming to improve efficiency and reliability. Considering the studies mentioned above, this paper aims to add value by directly linking the dielectric performance and material parameters to the accuracy of instrument transformers (ITs). Specifically, the focus is on the resin used in capacitive low-power voltage transformers (LPVTs). A comprehensive set of dielectric tests, detailed in the following sections, was conducted on the resin to evaluate its performance. Subsequently, the relationship between the resin’s properties and the accuracy of LPVTs is analyzed, with the results clearly demonstrating the extent of their influence.

The remainder of the paper is structured as follows: Section 2 introduces the use case, providing a detailed background and describing the items under test. Section 3 outlines the experimental procedures and the measurement setup employed. Section 4 presents and discusses the results obtained. Finally, Section 5 summarizes the conclusions derived from this study.

## 2. Use Case

### 2.1. Low-Power Voltage Transformers

LPVTs are an advanced class of instrument transformers designed to meet the demands of modern power systems. Unlike traditional inductive voltage transformers, LPVTs employ capacitive, resistive, or mixed voltage-dividing elements to step down medium and high voltages for measurement and control. This design offers significant advantages, including compactness, lighter weight, and reduced material usage, making them more cost-effective and environmentally friendly. LPVTs are particularly suited for applications in smart grids and digital substations due to their enhanced accuracy over a wide frequency range, excellent transient response, and immunity to electromagnetic interference. Furthermore, LPVTs are inherently safer to operate as they work with low energy levels, minimizing the risk of hazards. Their ability to provide accurate and reliable voltage measurements, even in the presence of harmonics and disturbances, makes them a critical component in the evolving landscape of intelligent power systems. LPVTs are standardized by the IEC 61869 series, within which document [19] fixes the general requirements, while document [20] is completely dedicated to them. In terms of accuracy, the documents provide parameters and limits based on the accuracy class of the device. However, there is no mention about the materials or the properties to be used by manufacturers during the construction of the devices.

Many manufacturers exploit the insulation properties of the resin that shield the LPVT as part of the capacitance of the divider. Then, the LPVT case becomes a crucial component, and its performance on the overall accuracy of the device must be evaluated.

### 2.2. The Goal

**Figure 1:**
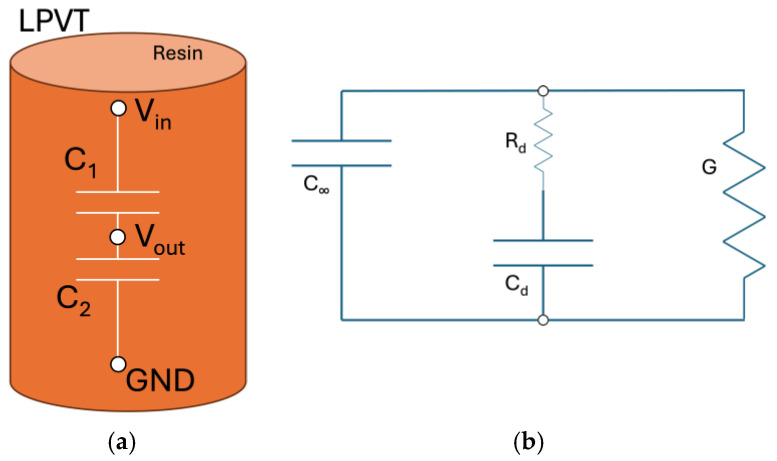
Ideal (**a**) schematic of the capacitive voltage divider technology and the real schematization of a capacitor (**b**).

The goal of this paper is to link the dielectric properties of the LPVT with the insulating material, the resin, used to build such a device. To this purpose, it is useful to introduce, with the help of Figure 1, the ideal schematic of an LPVT (Figure 1a) and the real model of a single capacitor (Figure 1b).

Of course, the ideal LPVT (Figure 1a) consists of two capacitors, $C_{1}$ and $C_{2}$, creating the well-known voltage divider configuration. $C_{1}$ is the smaller capacitance (depending on the desired voltage ratio to be achieved), and it is subjected to the major voltage drop compared to $C_{2}$. In the ideal case, the divider is not influenced at all by frequency, temperature, and other external influences. The picture is, then, completed with the input $V_{in}$, output $V_{out}$, and ground terminals GND. On the other hand, representing the real configuration (Figure 1b) is much more complex; in fact, any capacitor can be modeled as in the picture, where, in addition to the pure capacitance (*C_∞_*) related to the electronic and atomic polarization, we need to consider two additional branches. One is relevant to the dipolar polarization, where a capacitance *C_d_*, related to the dipolar polarization, is in series with a resistance *R_d_*, related to the hysteresis losses. The other one is characterized by a conductance *G*, considering the conduction phenomenon occurring throughout the dielectric. While conductance is negligible at power frequencies (50 or 60 Hz), this is not the case with the RC branch, representing the dipolar polarization, which is the main polarization mechanism occurring at power frequencies. All the components within the circuit reported in Figure 1b are strictly related to the dielectric material properties, being related to the complex permittivity ε and its conductivity through Equation (1):

(1) $$C=\varepsilon \cdot \frac{S}{d}$$

where *ε* is the complex permittivity, *S* is the surface, and *d* is the thickness of the insulation system through which the electric field is applied.

Opposite to the electronic and atomic polarization mechanism, the dipolar polarization is strongly influenced by temperature. Thus, it is crucial to monitor the modifications with the temperature of the IT capacitance ratio during its operation time. Indeed, how much the quantity is modified with temperature is strongly related to the material properties and local environmental conditions such as the presence of moisture contaminants. Consequently, it may be the case that the values related to the two reference capacitances within the measurement transformer may vary differently, possibly leading to inaccurate measurements.

## 3. Experimental Activity

### 3.1. Materials

Epoxy resins used within measurement transformers can vary significantly based on their specific compositions and the processes they undergo before installation. These variations include differences in the base polymer matrix, such as the chemical structure of the resin. The base resin is usually insufficient to withstand the environmental stress during the application times. For this reason, the types and quantities of additives employed can further distinguish different epoxy resin formulations. Additives might include hardeners, fillers, plasticizers, and other modifiers, each contributing unique properties to the final material, including permittivity [21] and space charge [22,23].

In this article, the authors mainly focus on the impact of curing time on the properties of the resin. Curing conditions refer to the duration, temperature, and method used to harden the resin, which directly impacts its mechanical, thermal, electrical, and chemical properties.

**Figure 2:**
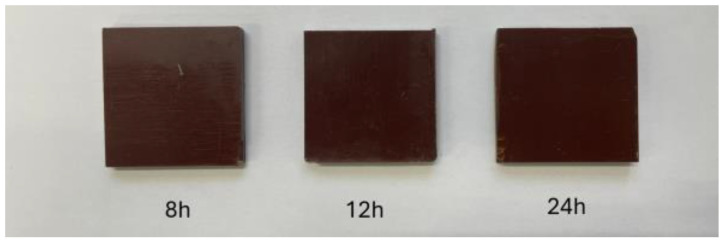
Photograph of the samples investigated. From the left, epoxy cured for 8 h, 12 h, and 24 h.

To investigate the impact of curing time on the chosen electrical properties, specimens used in this work are industrial-scale epoxy resins featuring the same polymer matrix with different curing times, i.e., 8 h, 12 h, and 24 h. By visual inspection, no particular differences may be undermined among the three curing times, possibly due to the other additives present within the base compound (Figure 2).

The samples were cut into 4 × 4 cm square shapes with a thickness of ~4 mm. The selection of this size is primarily influenced by the necessary dimensions of the dielectric analyzer sample cell. Before measurement, specimens were cleaned with isopropyl alcohol and put under vacuum at 50 °C for at least 8 h to remove any volatile products and bonded moisture.

Note, this paper exploits one type of resin, but the goal is not to quantify its specific goodness. This paper aims to highlight the strict link between the material and the accuracy of the ITs, increasing the consciousness of the users and the manufacturers while dealing with such devices.

### 3.2. Accelerated Aging

During the application life of the instrument transformers, epoxy resins may be modified by the effect of environmental stress, mainly high temperatures and ambient moisture. This may lead to the modification of the dielectric property of the resin [22,24]. To investigate the performance modification of the polymeric materials, epoxy resin samples underwent two different aging conditions aiming at simulating the conditions the measurement transformer faces during its application. Two conditions were considered in this work:

- Thermal aging at 90 °C up to 1000 h with withdrawal times of 250 h to simulate harsh dry conditions.
- Combined aging at 90 °C with 80% RH moisture up to 1000 h with withdrawal times of 250 h to simulate harsh moisture conditions.

Thermal aging was performed in an air-ventilated oven, while combined aging was performed in a Climatic Chamber. After withdrawal, experimental tests were conducted on the specimen.

### 3.3. Dielectric Spectroscopy

As reported in Section 2, the key parameter to consider for the ITs is the reference capacitance, which is defined by means of the real part of permittivity [21]. Thus, we chose to investigate the trend of complex permittivity as a function of the treatment time by means of dielectric spectroscopy measurements on both unaged and aged samples.

To do so, a Novocontrol Alpha Dielectric Analyzer v2.2 was employed with the following test parameters:

▪ Applied voltage, rms: 200 V;
▪ Frequency range: 10^−1^ to 10^4^ Hz;
▪ Temperature range: −50 °C to 50 °C (with temperature steps of 25 °C).

The selected temperature range aligns precisely with the operational temperature spectrum of the measurement transformers analyzed in this study. This correspondence facilitates the identification of specific phenomena associated with temperature variations.

The maximum relative error of the instrumentation for the frequency region considered is equal to 0.2%.

## 4. Results and Discussion

### 4.1. Effect of Curing Time on Unaged Epoxy Resins

**Figure 3:**
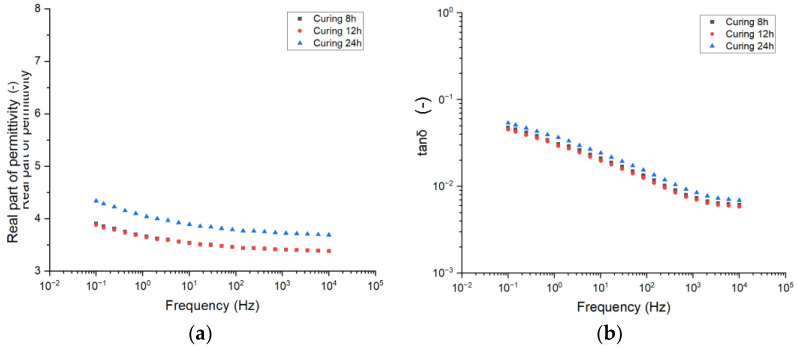
Complex permittivity as a function of frequency for different curing times. (**a**) Real part of permittivity. (**b**) Dissipation factor (tanδ).

In this first subsection, the effect of curing time on the dielectric properties of the epoxy resins is investigated. Figure 3 reports the trend of the real part of permittivity (a) and dissipation factor tanδ (b) as a function of frequency for the three different curing times considered.

With reference to the real part of permittivity, it is shown that 8 h and 12 h curing times do not modify the overall polarizability of the epoxy resin, leading to overlapped curves. On the contrary, the 24 h curing time causes an increase in the real part of permittivity. This may be related to either the formation of molecules with higher polarizability and dipolar momentum or an improved crosslinking network.

The first reason may be supported by the fact that a greater number of crosslinks generates a larger number of polar hydroxyether groups, thereby increasing overall material polarity [22]. Furthermore, in a crosslinked structure, the addition of more crosslinks within the polymer matrix decreases the average distance between crosslink points, leading to a reduction in free volume and an increase in material density. An increase in density is usually related to the increase in permittivity as it occurs in the case of linear low-density polyethylene (LLDPE) and high-density polyethylene (HDPE), featuring 2.2 and 2.4, respectively [23].

This behavior helps expand what is seen in the literature [17], where it is shown that, for a curing time up to 6 h, the real part of permittivity increases with the different curing times. In this study, the use of significantly longer curing times provides insight into how this dependence evolves with extended curing periods.

With reference to the dissipation factor (Figure 3b), it is shown that the trend over frequency is the same among the different materials. Indeed, one can notice the presence of a peak related to interfacial polarization in the lowest frequency region. This peak is partly placed outside the recordable frequency region. This suggests that all the materials featuring the presence of huge interfaces are probably linked to additives, which are known to be present in high concentration inside epoxy resins for electrical applications. Focusing on the amplitude value, similarly to what has been seen for the real part of permittivity, a slight increase in the dielectric losses is recorded for the 24 h cured epoxy, suggesting that the curing time may lead to the formation of new species whose response to the external electric field leads to higher losses.

### 4.2. Effect of Temperatures on Unaged Epoxy Resins

**Figure 4:**
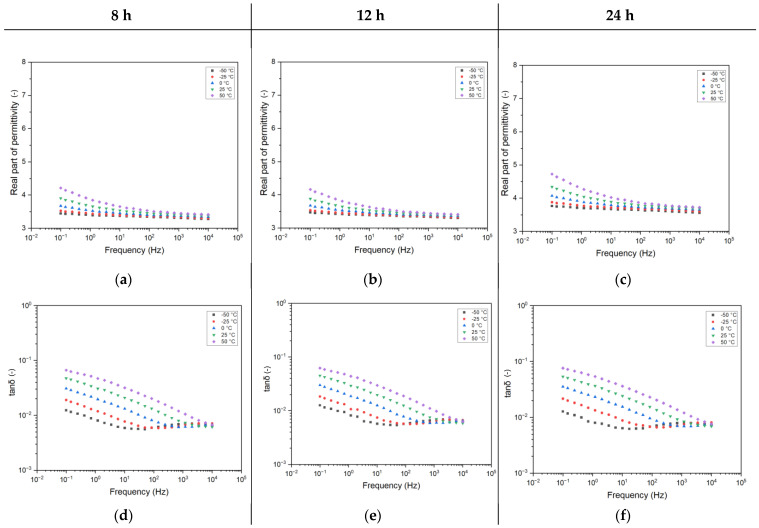
Complex permittivity as a function of frequency and testing temperatures for the different curing times. (**a**–**c**) Real part of permittivity. (**d**–**f**) Dissipation factor (tanδ). Curing times 8 h (**a**,**d**), 12 h (**b**,**e**), 24 h (**c**,**f**).

Since instrument transformers can operate at different temperatures based on environmental conditions, it is important to account for the resulting changes in capacitance. Figure 4 shows the trend of the real part of permittivity (a, b, c) and dissipation factor (tanδ) (d, e, f) as a function of frequency for different testing temperatures. Here, again, one can notice that, when varying the testing temperatures, practically no difference is recorded for the first two curing times (8 h and 12 h). On the contrary, the real part of permittivity for the 24 h cured epoxy shows a shift towards higher values (+12%). However, the variations with temperature increments are almost identical among all three samples. This implies that, while a longer curing time raises the baseline permittivity, the way permittivity changes with temperature remains consistent regardless of curing duration.

Normally, by increasing the testing temperature, the complex permittivity trend over frequency is shifted towards higher frequencies, as per theoretical behavior [21]. This permits a deeper investigation of the dielectric relaxation mechanisms within the polymer matrix, obtaining, e.g., the activation energy of the relaxation process. The results depicted in Figure 4 validate this behavior.

In particular, the analysis at very low temperatures (−50 °C) causes the appearance, in the high-frequency region, of the β peak, which was initially hidden at room temperature due to peak shifting. This peak is probably linked to the dipoles present in the epoxy resin, whose chemical formula is usually characterized by the presence of polar atoms [22].

Increasing testing temperatures also helps us understand the amplitude of the interfacial-related peak described in the previous section. As a result, at 50 °C, the entire dielectric spectrum is ruled by this interfacial peak, confirming the presence of huge interfaces within the polymer matrix, as already described above.

Contextually, the real part of permittivity shows an increasing trend in lowering the frequency, which is quite typical as per the dielectric relaxation theory [21]. This trend is, indeed, likely to be related to the right branch of a flex, which is, even for the highest temperature considered here, out of the measured frequency range of the instrumentation.

### 4.3. Effect of Aging on Epoxy Resins

#### 4.3.1. Aging Modifications at Room Temperature

**Figure 5:**
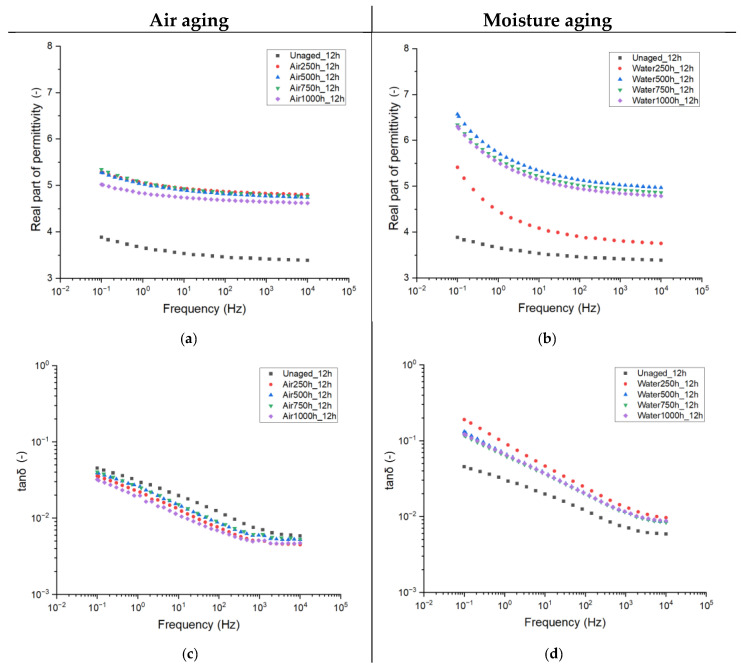
Complex permittivity as a function of frequency for different aging times for the 12 h cured resin. Air-only aging (**a**,**c**) and moisture aging (**b**,**d**).

Figure 5 illustrates the trend of complex permittivity as a function of frequency for materials aged with (a, b) and without (c, d) moisture. For the purposes of this study, the frequency-dependent results for epoxy resin cured for 12 h are discussed. In the following, a comparative data analysis will consider all the tested curing times.

It can be observed that, depending on the aging parameters, different modifications in complex permittivity are recorded.

With reference to in-air aging, ε’ at 50 Hz is seen to increase from ~3.5, a typical value for epoxy resins, to around 5.2 after the initial aging period. Further aging does not alter this electrical quantity, which remains constant up to 1000 h, with an almost negligible decrease recorded thereafter. Conversely, aging with moisture depicts completely different behavior. Initially, the first aging period (250 h) causes a slight increase in ε’ to about 4, while further aging (>500 h) raises it to approximately 6.5 at low frequencies, with minimal changes over time. Therefore, it can be concluded that, despite both the aging treatments leading to the increase in the real part of permittivity, moisture absorption within the insulating material causes the biggest increase in the property. A possible reason for that lies in the fact that water is an extremely polar molecule, and its interaction with the polymer matrix leads to an increase in the overall polarity of the epoxy.

With reference to the dielectric losses, the dissipation factor (tanδ) also records modification depending on the aging conditions. For the treatment with air only, one can observe a decrease in tanδ with aging, followed by little fluctuations. On the other hand, water treatment causes, as expected, an increase in the dielectric losses, probably due to the higher polarity of absorbed moisture. By considering the trend of property with frequency, a steeper increase in lowering the frequencies is recorded for the water treatment, possibly related to the higher conductivity of water molecules.

#### 4.3.2. Effect of Testing Temperatures on Aged Samples

In the following, the trend of complex permittivity for the 500 h and 1000 h aged samples are reported as a function of different testing temperatures. Such an analysis is significant, as complex permittivity varies as a function of temperature due to the modification of the activation energy of the dielectric relaxation process [21]. Moreover, as the ITs are usually placed outdoors, the modification of temperature may also affect the insulation system of the transformer and modify the capacity ratio, causing misleading measurements.

**Figure 6:**
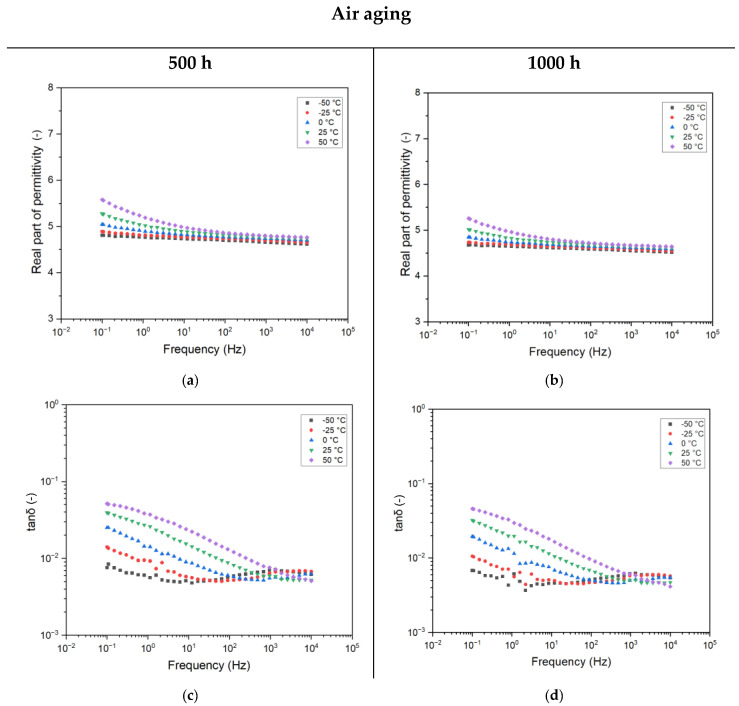
Complex permittivity as a function of frequency for different testing temperatures for the 12 h cured and aged resin: 500 h (**a**,**c**) and 1000 h (**b**,**d**) of aging.

From Figure 6, it is evident how the trend of frequency and temperature looks to be invariant with aging. What changes is the amplitude of the recorded electrical property. In particular, the value of real part of permittivity for the 500 h aged sample is increased from 4.2, of the unaged sample, to 5.6 (+33%) at 0.1 Hz and 50 °C. In terms of dissipation factor (tanδ), the modification may be considered negligible, as already discussed in the previous section. Further aging causes the downward shifting of the complex permittivity. This shifting is valid for all the testing temperatures, suggesting that, despite a slightly lower polarizability of the epoxy, the activation energy of the molecules within the matrix is almost constant. Similarly, the dissipation factor (tanδ) is almost halved by increasing the aging time.

**Figure 7:**
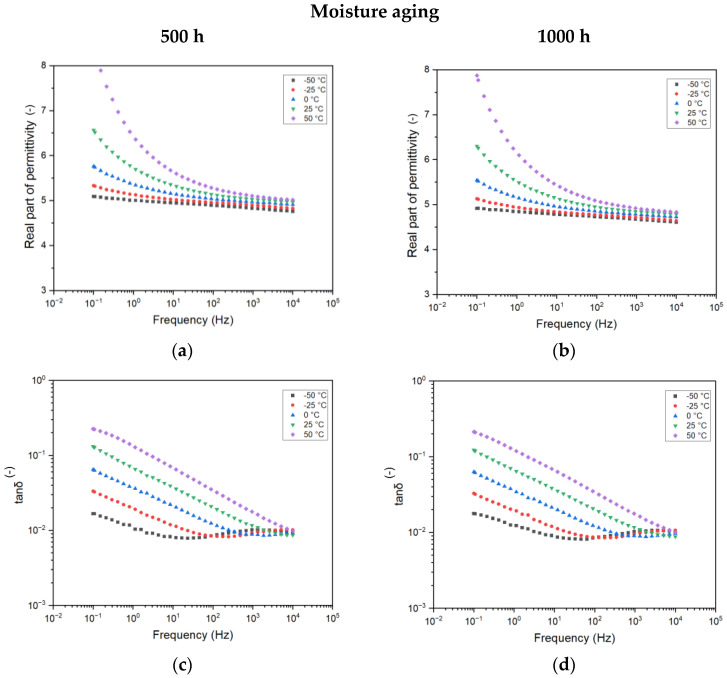
Complex permittivity as a function of frequency for different aging times for the 12 h cured resin aged under moisture: 500 h (**a**,**c**) and 1000 h (**b**,**d**) of aging.

With reference to the moisture-treatment-related materials (Figure 7a,b), one can notice similar behavior with frequency when varying the temperatures of both the parameters investigated. Nonetheless, the amplitude of the variation is significantly different, increasing up to 8.5 (120%) for the real part of permittivity and up to 3 × 10^−1^ (450%) for the tanδ. This increase is expected, as it may be directly linked to the presence of the more highly conductive water molecules permeated during the aging process within the resin, as reported in the previous section. Additionally, different testing temperatures help to reveal the extent of this process. Higher temperatures can accelerate the movement and interaction of water molecules within the material, thereby amplifying the changes in electrical properties and highlighting the conductive nature of water molecules.

### 4.4. Modifications of Dielectric Properties at 50 Hz

**Figure 8:**
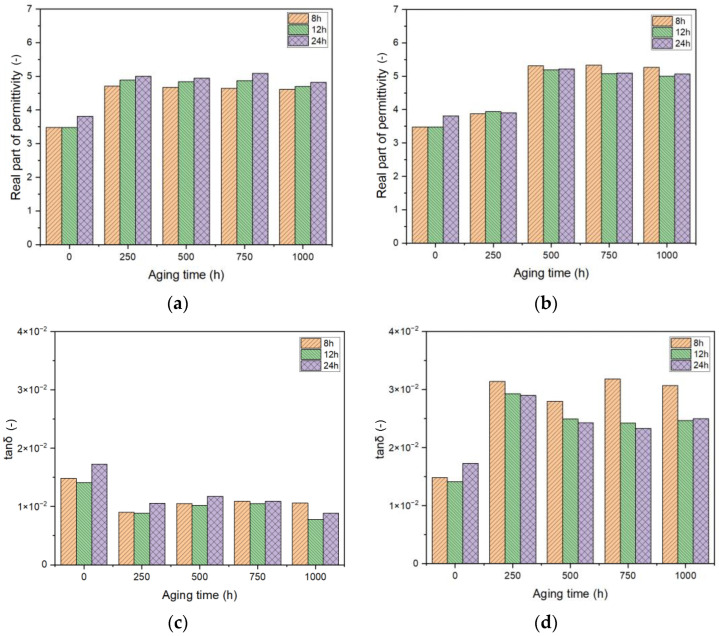
Complex permittivity at 50 Hz as a function of frequency for different aging times for the 12 h cured resin. Air-only aging (**a**,**c**) and moisture aging (**b**,**d**).

Aiming at better investigating the effect of aging time and bonded moisture as a function of curing time, we chose to report the values of the real part of permittivity (Figure 8a,b) and dissipation factor (tanδ) (Figure 8c,d) at 50 Hz, which is the operating frequency of measurement transformers. This would help highlight the differences and modifications that the reference capacitance may face during the application life of the ITs.

At first glance, it is possible to notice that the trend of aging is different among the two aging conditions (air and moisture) for both the analyzed parameters. The real part of permittivity is seen to increase by almost 75% from the initial value for both conditions

Bigger differences are seen in the case of tanδ, as expected, with bonded moisture causing the property to increase substantially, reaching up to twice its initial value

Focusing on the air-only treatment, Figure 8a exhibits an increase in permittivity with aging for all curing times. Consistently, the curing times follow the increase in permittivity values. As a matter of fact, the epoxy resin cured for 24 h exhibits the highest permittivity values across all aging times, suggesting more significant changes in its electrical properties compared to resins cured for shorter periods. Resin cured for 12 h shows intermediate permittivity values, while the resin cured for 8 h has the lowest permittivity values, indicating that shorter curing times result in less significant changes over time. A comparative analysis at different aging intervals highlights the influence of the initial curing process on the long-term electrical behavior of the epoxy resin.

This behavior changes by considering the moisture aging (Figure 8b). Here, treatments longer than 500 h are needed to highlight significant modifications in the property. The values are then stabilized up to 1000 h. Interestingly, opposite from what is reported above, the resin cured for 8 h exhibits the highest permittivity values throughout all aging times, followed by the resin cured for 12 h, with the resin cured for 24 h showing the lowest values. This behavior may possibly be linked to the higher free volume present in the case of 8 h cured resin, permitting an easier permeation of water in between the polymer chains.

Also, considering the dissipation factor (Figure 8c,d), the two aging conditions depict different behaviors, highlighting a strong increase in the dielectric losses in the case of moisture aging (Figure 8d).

In air, the unaged samples depict a higher loss tangent for all curing times, with the 24 h curing time showing the highest initial value. With aging, the property is almost halved and the loss tangent values for all curing times tend to stabilize, though slight variations are observed. At each aging interval, the loss tangent values differ based on the curing time, as seen for the real part of permittivity. However, no monotonic behavior may be highlighted since the variations are very limited, leading to the conclusion that the impact of curing time of the epoxy resins for *in-air* aging is almost negligible.

With moisture, the behavior results are very different. The first aging time causes an increase in tanδ, doubling the initial value. Further aging causes a slight decrease in the property. For example, after 250 h, the 24 h cured resin shows a noticeable decrease, while the other curing times exhibit less pronounced changes. As reported in the previous section, the increase in tanδ due to the permeation of moisture may be linked to the high polarity of water molecules, which are characterized by high permittivity (~80) and dielectric losses. As a result, the overall electrical performance of the epoxy resin is decreased, leading to the high real part of permittivity and dielectric losses, which may lead to the different distribution of electric fields within the insulation and unexpected energy losses.

Epoxy resins cured for 8 h exhibit the highest dielectric losses, indicating that shorter curing durations may lead to increased energy dissipation within the material. Conversely, those cured for 12 h show intermediate dielectric losses, suggesting a more balanced performance in terms of energy dissipation. Eventually, epoxy resins cured for 24 h present the lowest dielectric losses, highlighting the benefit of extended curing times in achieving superior dielectric properties and more efficient insulation performance. Indeed, a longer curing time leads to the formation of a more stable and robust molecular structure within the epoxy resin, as proved by the results reported in this work [25].

This indicates that longer curing times improve the electrical insulation performance of epoxy resins, making them more suitable for applications where minimizing dielectric losses is necessary [26,27].

### 4.5. Impact on Instrument Transformer Accuracy

The presented results provide clear insights into the dielectric property changes on the resin that have a consequential impact on the ITs’ accuracy. In fact, considering that ITs can exploit the resin as one of the capacitors, as shown in Figure 1, the capacitance value (see Equation (1)) is affected and impacts the accuracy of the IT. This clearly starts from the ideal voltage divider relation for a capacitive divider:

(2) $$V_{out}=V_{in}\frac{C_{1}}{C_{1}+C_{2}}=\frac{\varepsilon_{1}\cdot \frac{S_{1}}{d_{1}}}{\varepsilon_{1}\cdot \frac{S_{1}}{d_{1}}+\varepsilon_{2}\cdot \frac{S_{2}}{d_{2}}}$$

where $\varepsilon_{1}$, $S_{1}$, and $d_{1}$ refer to $C_{1}$, $\varepsilon_{2}$, and $S_{2}$, and $d_{2}$ refers to $C_{2}$. Therefore, when the dielectric properties of $C_{1}$ change, while $C_{2}$ remains unaffected because of a different technology, the input/output ratio changes. This has a direct effect on the accuracy of the IT. This is confirmed by the results:

▪ The prolonged curing time of the resin, without any other external influences, causes a variation in the real part of the permittivity of more than 10% over the entire frequency range.
▪ When the temperature is changed, the real part of the permittivity is again affected, and it increases when the temperature increases. The variation is smoothed as the frequency increases and the curing time does not contribute to such a variation.
▪ When including humidity in the ageing process, the real part of the permittivity shows another behavior. In fact, it has been demonstrated that the presence of humidity produces a more significant variation compared to the aging case without humidity. Variations up to 120% are documented and the consequential impact on the IT accuracy becomes undeniable.

To further clarify the effect on the transformers’ accuracy, let us introduce the definition of ratio error, as defined in [20]:

(3) $$\varepsilon =\frac{K_{r}U_{s}-U_{p}}{U_{p}}100\%$$

where $K_{r}$ is the rated transformation ratio, and $U_{p}$ and $U_{s}$ are the rms values of the primary and the secondary voltage, respectively. Consequently, if a change in the transformer ratio is proved (see Equation (2)) by the above-reported results, Equation (3) will change by the same amount. To quantify such an effect, consider that changes from a few percent to more than 120% have been reported. These values must be compared to the accuracy class limits (see [19,20]), which include ratio error variations from 0.1% to 3%.

## 5. Conclusions

Epoxy resins used in measurement transformers can vary in terms of types (e.g., base polymer matrix and employed additives) and, within the same type, also in terms of curing and post-treatment conditions. These parameters deeply influence the final electrical performance of the material and, consequently, the final measurement accuracy.

This study demonstrates that both curing time and aging significantly affect the real part of permittivity of epoxy resin. Longer curing times result in greater changes in permittivity over time, while shorter curing times lead to more pronounced variations. Additionally, aging with moisture has a substantial impact on permittivity, with increased water permeation through the resin intensifying these changes. Indeed, water molecules are an extremely polar species, and they proved to significantly impact the dielectric response, leading to a general depletion of the epoxy properties. In addition, the curing time of the different epoxies affected the modification of the dielectric properties over time, and it was likely attributed to the different permeation rates of moisture due to the changes in the free volume. Specifically, a shorter curing time (8 h) results in a material with lower density, thereby allowing water to permeate more easily. This moisture ingress is proved to alter the capacity ratio of the ITs, leading to potential measurement inaccuracies.

Furthermore, epoxy resins are seen to have consistent temperature dependence, both in dry and moisture aging conditions. This dependence turned out to be invariant with the curing time differences.

In conclusion, the curing time of the resin and the external temperature can significantly influence the real part of permittivity and, thus, the measuring capacitance through the transformer. If not properly addressed, these influence factors can modify the accuracy performance of the devices. Therefore, a preliminary characterization vs. influence quantities or a proper selection of the insulation material are two examples of countermeasures to avoid unexpected results.

Future work on this topic will include a deeper investigation into the quantity of moisture permeated within the material by means of physical–chemical analyses such as FTIR and Karl–Fischer methods in the framework of deriving a correlation between the environmental stresses and conditions with the instrument transformers’ measurements.

## Data Availability

Data available upon request.
